# Comparative Proteomic Analysis of Huh7 Cells Transfected with Sub-Saharan African Hepatitis B Virus (Sub)genotypes Reveals Potential Oncogenic Factors

**DOI:** 10.3390/v16071052

**Published:** 2024-06-29

**Authors:** Kiyasha Padarath, Aurélie Deroubaix, Previn Naicker, Stoyan Stoychev, Anna Kramvis

**Affiliations:** 1 Hepatitis Virus Diversity Unit, Department of Internal Medicine, School of Clinical Medicine, Faculty of Health Science, University of Witwatersrand, 7 York Road, Parktown, Johannesburg 2193, South Africaaurelie.deroubaix@wits.ac.za (A.D.); 2 Life Sciences Imaging Facility, Faculty of Health Sciences, University of the Witwatersrand, 7 York Road, Parktown, Johannesburg 2193, South Africa; 3 Future Production Chemicals, Council for Scientific and Industrial Research, Pretoria 0184, South Africa; pnaicker1@csir.co.za; 4 ReSyn Biosciences, Johannesburg 2000, South Africa; sstoychev@resynbio.com; 5 Evosep Biosystems, 5230 Odense, Denmark

**Keywords:** hepatitis B virus, (sub)genotypes, hepatocellular carcinoma, viral oncogenesis, oncogenic pathways

## Abstract

In sub-Saharan Africa (SSA), the (sub)genotypes A1, D3, and E of the hepatitis B virus (HBV) prevail. Individuals infected with subgenotype A1 have a 4.5-fold increased risk of HCC compared to those infected with other (sub)genotypes. The effect of (sub)genotypes on protein expression and host signalling has not been studied. Mass spectrometry was used to analyse the proteome of Huh7 cells transfected with replication-competent clones. Proteomic analysis revealed significantly differentially expressed proteins between SSA (sub)genotypes. Different (sub)genotypes have the propensity to dysregulate specific host signalling pathways. Subgenotype A1 resulted in dysregulation within the Ras pathway. Ras-associated protein, RhoC, was significantly upregulated in cells transfected with subgenotype A1 compared to those transfected with other (sub)genotypes, on both a proteomic (>1.5-fold) and mRNA level (*p* < 0.05). Two of the main cellular signalling pathways involving RHOC, MAPK and PI3K/Akt/mTOR, regulate cell growth, motility, and survival. Downstream signalling products of these pathways have been shown to increase MMP2 and MMP9 expression. An extracellular MMP2 and MMP9 ELISA revealed a non-significant increase in MMP2 and MMP9 in the cells transfected with A1 compared to the other (sub)genotypes (*p* < 0.05). The upregulated Ras-associated proteins have been implicated as oncoproteins in various cancers and could contribute to the increased hepatocarcinogenic potential of A1.

## 1. Introduction

Despite the availability of an effective vaccine, hepatitis B virus infections remain hyperendemic in the sub-Saharan Africa (SSA) region. HBV, one of the seven known oncogenic viruses, is responsible for the high incidence rate of hepatocellular carcinoma (HCC) in SSA. In 2020, SSA had the fourth-highest number of diagnosed primary liver cancer (PLC) cases worldwide, of which 77% were HCCs (varying from 67% to 88% by region) [1]. This cancer accounts for 8.3% of the deaths caused by all cancers globally, being the third leading cause of worldwide cancer death [2,3]. It is estimated that, by 2025, more than one million individuals will develop liver cancer annually [4]. In less developed countries, HCC occurs in chronic viral hepatitis patients more frequently than in more developed Western countries (e.g., 53% of the cases in South Africa [5] compared to only 9.5% in the U.S. [6]). 

In addition to the hyperendemic occurrence of HBV in SSA, genetic variation in HBV can influence the clinical manifestation of infection. HBV reverse transcriptase lacks a proofreading function, which allows the virus to accumulate genetic errors over time, leading to sequence heterogeneity [7,8,9,10]. This genetic diversity has resulted in at least nine genetically distinct HBV genotypes, designated A to I, and a tenth putative, genotype J [8,11,12,13], with >7.5% intergroup nucleotide divergence [12,14,15,16]. The nine genotypes are further classified into at least 35 subgenotypes, based on a nucleotide divergence of 4–8% [11,17,18,19]. The genotypes and certain subgenotypes have distinct geographical distributions and are important in both the clinical manifestation of infection, response to antiviral therapy, and the development of HCC. In SSA, the most prominent (sub)genotypes are A1, D3, and E [20,21].

Subgenotype A1, which prevails in Southeastern SSA, has a higher hepatocarcinogenic potential, with a 4.5-fold increased risk of HCC development and an earlier age of diagnosis in patients infected with subgenotype A1 compared to other HBV genotypes [12,21,22]. Subgenotype D3 is associated with high chronicity, which can lead to cirrhosis and HCC, whilst genotype E is associated with significantly higher viral loads and a higher frequency of HBeAg positivity [8,19,23,24,25,26]. Subgenotype A2, which prevails outside SSA and has been used as a comparison for (sub)genotypes prevailing in SSA, has a different natural history to A1, D3, and E [27]. In a case–control study, comparing individuals infected with subgenotypes A1 and A2, and genotype D, respectively, it was shown that, regardless of the age group, the frequency of HBeAg positivity was higher in individuals infected with subgenotype A2 compared to the other (sub)genotypes [28]. This difference reached statistical significance in individuals younger than 30 years of age [22,28]. The reason for this is that the only mutations that can influence HBeAg expression in strains isolated from individuals infected with subgenotype A2, which has 1858C in the precore region, are 1762T/1764A in the basic core promoter (BCP). This mutation only decreases HBeAg levels [29]. On the other hand, subgenotype A1 strains, which also have 1858C, can develop additional mutations in the Kozak sequence within the BCP (1809–1812) [30] and the G1862T in the precore [31,32], which further decreases HBeAg levels [22] and thus accounts for the higher frequency of HBeAg negativity observed in individuals infected with subgenotype A1. Subgenotype D3 and genotype E, which both have 1858T, can develop the classic G1896A mutation, which abrogates HBeAg expression as a result of a stop codon [33].

Proteomic studies have aimed to describe global changes in host cells following HBV infection [34,35,36]. Previous studies have shown that the protein expression alterations between HBV genotypes B and C are involved in several different biological processes, including translation, oxidation–reduction, intracellular transport, the establishment of protein localisation, protein transport, and translational elongation, the pathogenesis of HBV-induced HCCs associated with different molecular mechanisms [34,37,38]. Currently, little is known about the differences in the specific biological pathways induced by the different HBV genotypic backgrounds found in SSA. Therefore, this study provides the first report of the molecular differences at the proteome level between Huh7 cells transfected with the SSA (sub)genotypes A1, D3, and E, and subgenotype A2, which prevails outside of Africa. Understanding the role of genotypic variation in pathogenesis is important and will provide further insight into patient outcomes, chronicity, infectivity, response to therapeutic approaches, and improve the early detection and screening of HCCs in SSA.

## 2. Materials and Methods

### 2.1. Plasmid Constructs

A collection of 1.28-mer replication-competent plasmids for wild-type (sub)genotypes A1 (KM519453), A2 (KM519454), D3 (KM519455), and E (PP790594) were constructed and were able to replicate and express proteins in vitro [39,40]. Ethics approval to perform this study was obtained from the University of the Witwatersrand Research Ethics Committee (Medical), Johannesburg, South Africa (HREC: W-CBP-210416-01).

### 2.2. Cell Line and Transfections 

Huh7 cells were transfected as previously described [39]. Twenty-four hours before transfection, 3 × 10^6^ Huh7 cells were plated into each of the 10 cm culture dishes for mass spectrometry, whilst 2 × 10^5^ Huh7 cells were plated in each of the 12 well plates containing either coverslips for indirect immunofluorescence or no coverslips for the real-time polymerase chain reaction (RT-PCR) (viral load/RNA expression) or enzyme-linked immunosorbent assay, ELISA, and then incubated overnight at 37 °C in a humidified incubator containing 5% (*w*/*v*) CO_2_. For each transfection experiment, an eGFP control was included in triplicate to measure the transfection efficiency 1 day after transfection by determining the number of cells that had been successfully transfected. This was measured using the FLoid™ Cell Imaging Station (Thermofisher, Waltham, MA, USA).

### 2.3. Mass Spectrometry Analysis

#### 2.3.1. Cell Lysis and Protein Preparation

Five days post-transfection, the cells were washed 3 times with PBS. Aliquots of 0.5 million cells were prepared and pelleted at 300× *g* for 10 min. The supernatants were removed and cell pellets were stored at −80 °C until further use. For protein preparation, the cell pellets were thawed on ice and resuspended in 200 μL of lysis buffer (1% SDS, 50 mM Tris-HCl pH 8.0) per pellet. Protein was extracted from the cell pellets using a PIXUL multi-sample sonicator (Active Motif, Carlsbad, CA, USA). The sonication settings were as follows: Pulse = 50; PRF = 1; Process Time = 30; and Burst = 20. Thereafter, the lysates were incubated with 25 units of benzonase, and MgCl_2_ to a final concentration of 2 mM, at 37 °C for 30 min. Next, the samples were centrifuged at 15,000× *g* to clear cell debris. The supernatants were then collected, and the concentrations were determined using a BCA assay. Protein solutions were reduced using DTT to a final concentration of 10 mM for 30 min at 37 °C, and then alkylated using IAA to a final concentration of 40 mM for 30 min in the dark.

#### 2.3.2. Sample Clean-Up and Digestion

All experiments were performed with a KingFisher™ Flex (Thermo Fisher Scientific, Waltham, MA, USA) magnetic particle processing robot, as previously described [41]. The KingFisher™ Flex system was configured for automated HILIC protein clean-up and on-bead Trypsin digestion. In brief, deep 96 well plates were loaded in each carousel position, with each plate filled as follows: (1) 96 well tip heads (Thermo Fisher Scientific, Waltham, MA, USA); (2) 10 µL of 20 mg/mL hyper porous magnetic MagReSyn HILIC microspheres (ReSyn Biosciences, Pretoria, South Africa) in 20% EtOH and 180 µL of Equilibration Buffer (100 mM NH_4_Ac, 15% MeCN pH 4.5); (3) Equilibration Buffer (500 µL); (4) Protein extract mixed 1:1 with Binding buffer (200 mM NH_4_Ac, 30% MeCN pH 4.5) to a final volume of 100 µL; (5) 500 µL of 95% MeCN (wash 1); (6) 500 µL of 95% MeCN (wash 2); (7) 200 µL of 50 mM Ammonium Bicarbonate (pH 8.0); and (8) Promega Sequencing Grade Trypsin for an enzyme/protein ratio of 1:10. The Bindit programme was then run, with the magnetic pins transferring the magnetic HILIC beads from position 2 to 8 and, in the process, binding proteins, washing off SDS and other contaminants, and finally generating peptides ready for LC–MS analysis following the on-bead Trypsin digest.

#### 2.3.3. LC–MS Data Acquisition

Following HILIC, the peptide samples were vacuum dried and then resuspended in 2% MeCN/0.2% FA. Analysis was performed on a Dionex Ultimate 3000 RSLC system coupled to an AB Sciex 5600 TripleTOF mass spectrometer (Sciex, Toronto, ON, Canada). The injected peptides were inline de-salted using an Acclaim PepMap C18 trap column (Thermofisher, Waltham, MA, USA) (75 μm × 2 cm; for 2 min at 5 μL/min, using 2% ACN/0.2% FA). The trapped peptides were gradient eluted and separated using a Waters Acquity CSH C18 NanoEase column (Waters^TM^, Milford, MA, USA) (75 μm × 25 cm, 1.7 µm particle size) at a flowrate of 0.3 µL/min with a gradient of 6–40% B over 60 min (A: 0.1% FA; B: 80% ACN/0.1% FA). For Sequential Window Acquisition of all Theoretical Mass Spectra (SWATH), precursor scans were acquired from 400 to 1100 *m*/*z* with an accumulation time of 50 milliseconds and fragment ions acquired from 200 to 1800 *m*/*z* for 48 variable-width precursor windows, with a 0.5 Da overlap between the windows and an accumulation time of 20 milliseconds per window.

#### 2.3.4. SWATH–MS Data Processing

SWATH data was processed using Spectronaut v17 software (Biognosys, Zurich, Switzerland). The default directDIA identification and quantification settings were used for data processing. Carbamidomethylation was added as a fixed modification, and N-terminal acetylation and methionine oxidation were added as variable modifications. Swiss-Prot Human sequences (downloaded on 12 December 2022 from www.uniprot.org) and common contaminating proteins was used as the search database. A q-value ≤ 0.01 cut-off was applied at the precursor and protein levels. Quantification was performed at the MS1 and MS2 levels. Label-free cross-run normalisation was employed using a global normalisation strategy. Candidate dysregulated proteins were filtered at a q-value ≤ 0.05, absolute fold change (FC) ≥ 1.5, and a minimum of two unique peptides identified.

#### 2.3.5. Mass Spectrometry Bioinformatics 

##### Data Visualisation

Volcano plots and heat maps were generated using https://www.bioinformatics.com.cn/, accessed on 12 March 2024. All Venn diagrams were drawn using Venny (Oliveros, J.C. (2007–2015)), an interactive tool was used for comparing lists with the Venn diagrams (https://bioinfogp.cnb.csic.es/tools/venny/index.html, accessed on 24 March 2023).

##### Pathway Analysis

A list of significantly differentially expressed proteins (*p*-value ≤ 0.05) was analysed using the Protein Analysis Through Evolutionary Relationships (PANTHER online: http://pantherdb.org, accessed on 23 March 2023) classification system [42]. PANTHER is a comprehensive system which combines gene function, ontology, pathways, and statistical analysis tools to allow for the analysis of a large volume of data [43]. The top 10 signalling pathways of subgenotype A1 compared to the untransfected cells, vector control, and each of the other (sub)genotypes A2, D3, and E, were categorised.

##### Gene-Set Enrichment Analysis (GSEA)

Gene-set enrichment analysis (GSEA) allows for the detection of modest but coordinated changes in the expression of protein-encoding genes involved in a common biological function. To determine the top exclusive oncogenic pathways amongst the various (sub)genotypes compared to the vector control, GSEA was performed by the R package clusterProfiler (version 4.3.3). The t-statistic mean of the genes was computed in Hallmark’s pathway using 4 replications per condition. The upregulated pathways were defined by a normalised enrichment score (NES) > 0. Gene sets with a *p*-value less than 0.05 were considered to be significantly enriched or depleted.

##### STRING Analysis

The STRING (Search Tool for the Retrieval of Interacting Genes/Proteins) is a curated knowledge database used for the illustration of predicted interactions of identified proteins and neighbour genes. The proteins that were significantly differentially expressed in subgenotype A1, relative to each of the other (sub)genotypes and the vector control, were processed in STRING version 12.0 (https://string-db.org/, accessed on 12 March 2024) to obtain medium-confidence interaction data (score ≥ 0.4). The PPI network was visualised using the Cytoscape 3.2.1 software (https://cytoscape.org/, accessed on 12 March 2024).

### 2.4. RNA Expression Levels

Total RNA was isolated from the Huh7 cells transfected with the various (sub)genotypes and controls (untransfected and vector controls) using the RNAeasy mini extraction kit (Qiagen, Hilden, Germany), according to the manufacturer’s instructions. An aliquot of 500 ng of RNA was reverse transcribed to generate cDNA using the SuperScript™ VI cDNA First-Strand Synthesis System synthesis kit (Invitrogen, Boston, MA, USA), as per the manufacturer’s instructions. A total of 5 µL of cDNA (1:10 dilution) was amplified using the SYBR^®^Green Realtime PCR Master Mix (Thermofisher, Waltham, MA, USA) and performed on a MyCycler Thermocycler (Bio-Rad Laboratories, Hercules, CA, USA). Thermal cycling conditions were as follows: Initial denaturation at 95 °C for 2 min, followed by 25 cycles of denaturation at 95 °C for 30 s, annealing at 55 °C for 30 s, elongation at 72 °C for 1 min, and a final elongation at 72 °C for 10 min. Three relative expression levels of the target genes were calculated using the ∆Ct method, with GAPDH as the control housekeeping gene. Primers for the qPCR are listed in the following Table 1.

### 2.5. Viral Loads 

Analysis of the viral loads was performed using the protocol described by Bhoola et al. [39]. On day 5 post-transfection, the total DNA was extracted from the harvested transfected Huh7 cells using the NucleoSpin^®^ Tissue kit (Macherey–Nagel, Nordrhein-Westfalen, Germany). Meanwhile, an aliquot of 5 mL of transfected medium that had been collected on day 3 or 5 was centrifuged at 22,000× *g* for 5 min at 4 °C, after which a 100 μL aliquot of the sample was adjusted with 10 μL of 10× Incubation Buffer (Roche Diagnostics, Basel, Switzerland), treated with 1 µL of 10 U/µL DNase I (Roche Diagnostics) and 10 µL 100 mg/mL RNase A (Fermentas Molecular Biology Tools, Waltham, MA, USA), and then incubated at 37 °C for 20 min. Subsequently, the reaction was terminated by adding 5 µL of 0.2 M EDTA, pH 8.0, and then incubating at 75 °C for 10 min. Thereafter, extracellular total DNA and intracellular total DNA were extracted using the NucleoSpin^®^ Tissue kit (Macherey–Nagel, Nordrhein-Westfalen, Germany). The PCR primers, HBV DNA F (5′-CGTGTGTCTTGGCCAAAATTCG-3′) and HBV DNA R (5′-CATCCAGCGATAACCAGGACAA-3′), with a FAM/NFQ labelled TaqMan^®^ MGB probe (5′-FAM-TCACTCACCAACCTCC-NFQ-3′), were used to quantify HBV DNA in the ABI 7500 Real-Time PCR system (Applied Biosystems by Life Technologies, Waltham, MA, USA), using the PCR thermal cycling conditions previously described [44]. A serial dilution of plasmid DNA containing a single genome of subgenotype A1, ranging from 1 × 10^4^ to 1 × 10^8^ copies/mL, was used as a template to generate the standard curve. The second WHO International Standard for Hepatitis B Virus DNA (National Institute for Biological Standards and Controls (NIBSC), Hertfordshire, UK), which has a final concentration of 1 × 10^6^ IU/mL, was used as the positive control and for calibration of the standard curve.

### 2.6. ELISA 

After 5 days post-transfection, the expression of MMP9, MMP2, and RHOC were analysed with various ELISA kits according to the manufacturer’s protocol. Intracellular proteins were harvested by sonication. The concentrations were determined using a standard curve generated using the manufacturer’s protocol.

1. MMP9—Human MMP-9 ELISA Kit (RK00217) (ABclonal, Woburn, MA, USA) https://abclonal.com/elisa-kits/HumanMMP-9ELISAKit/RK00217, accessed on 12 December 2023
2. MMP2—Human Matrix Metalloproteinase 2 ELISA Kit (MMP2) (RK00309) (ABclonal, Woburn, MA, USA) https://abclonal.com/elisa-kits/HumanMMP-2ELISAKit/RK00309, accessed on 12 December 2023
3. RHOC—Human Rho-related GTP-binding protein RhoC (RHOC) ELISA Kit (RK11965) (ABclonal, Woburn, MA, USA) https://ap.abclonal.com/elisa-kits/HumanRhorelatedGTPbindingproteinRhoCRHOCELISAKit/RK11965, accessed on 12 December 2023.

### 2.7. Immunofluorescence

The following protocols were adapted from Deroubaix et al. [45]. Cells cultured on coverslips in 12 well plates were washed 3 times with PBS and then fixed with 3.7% formaldehyde in 1x PBS for 10 min at room temperature. The fixed cells were again washed 3 times with 1x PBS and permeabilised with 0.1% Triton–PBS for 7 min, before being washed 3 more times with PBS. The cells were then incubated for 1 h at room temperature with 1% bovine serum albumin (BSA, Fraction V, Roche Diagnostics GmbH by Roche Applied Science, Basel, Switzerland) (diluted in PBS 1x). Next, the cells were incubated with the respective primary antibodies (Transfected cells: anti-HBV core protein rabbit DAKO [46], Agilent Technologies (Santa Clara, CA, USA), 1/1000, anti-RHOA/RHOC mouse, Invitrogen, 1/250) for 1 h at 37 °C. Then the cells were washed 8 times with PBS 1X and incubated with the corresponding secondary antibodies (Alexa-Fluor 488 labelled anti-mouse (1:1000, Invitrogen) and Alexa Fluor 546 labelled anti-rabbit (1:1000, Invitrogen)) for 1 h at 37 °C. Cells were washed 8 more times with 1x PBS. The DNA was stained with 4′,6-Diamidino-2-Phenylindole, Dihydrochloride (DAPI, 1 mg/mL, 1/2000, Sigma-Aldrich, St. Louis, MO, USA) for 10 min at room temperature in the dark. The coverslips were then mounted using Fluoromount™ Aqueous Mounting Medium (Sigma-Aldrich), sealed using a clear nail polish after 3 h, and then analysed by fluorescent microscopy the following day.

### 2.8. Microscopy and Image Analysis

Microscopy was performed using a Leica Microsystems THUNDER Imager Tissue Microscope (Wetzlar, Germany) equipped with a 63x objective (HC PL FLUOTAR 63x/1.30 OIL by Leica Microsystems, Wetzlar, Germany) and Leica Application Suite X (LAS X) software (version 1.4.6 28433). The exposure time for each channel (Alexa Fluor 488 (325.05 ms), Alexa Fluor 546 (475.95 ms), and DAPI (11.25 ms)) was kept constant for each condition ((sub) genotypes, untransfected cells, and controls) to obtain comparable results between the different experimental slides. The exposure time was determined by removing any background staining from the negative control for Alexa Fluor 448 and 546. After capturing the image, the Leica deconvolution software was used to process the images. Quantification of fluorescence was performed using ImageJ software (Fiji/Image J (version: ij153), accessed on https://imagej.net/Fiji/Downloads), as described by Deroubaix et al. [45]. Briefly, the intensity of fluorescence for RHOA/RHOC across the cytoplasm was measured, averaged across a minimum of 30 transfected cells per (sub)genotype, and then compared to the untransfected control. 

### 2.9. Data Statistical Analysis

Statistics were calculated using GraphPad Prism version 10.2.2.

## 3. Results

### 3.1. Comparison of the Proteome of Huh7 Cells Transfected with the SSA HBV Genotypes 

Following transfection of the Huh7 cells with replication-competent clones and a vector control, the proteome was analysed 5 days post-transfection. Using SWATH–MS, a specific variant of the data-independent acquisition (DIA) method, 3906 proteins and 36 107 peptides were identified in the Huh7 cells transfected with the different HBV (sub)genotypes (Figure S1). Principal component analysis (PCA) was able to differentiate the proteome of the cells transfected with individual (sub)genotypes (Figure S1).

**Figure 1:**
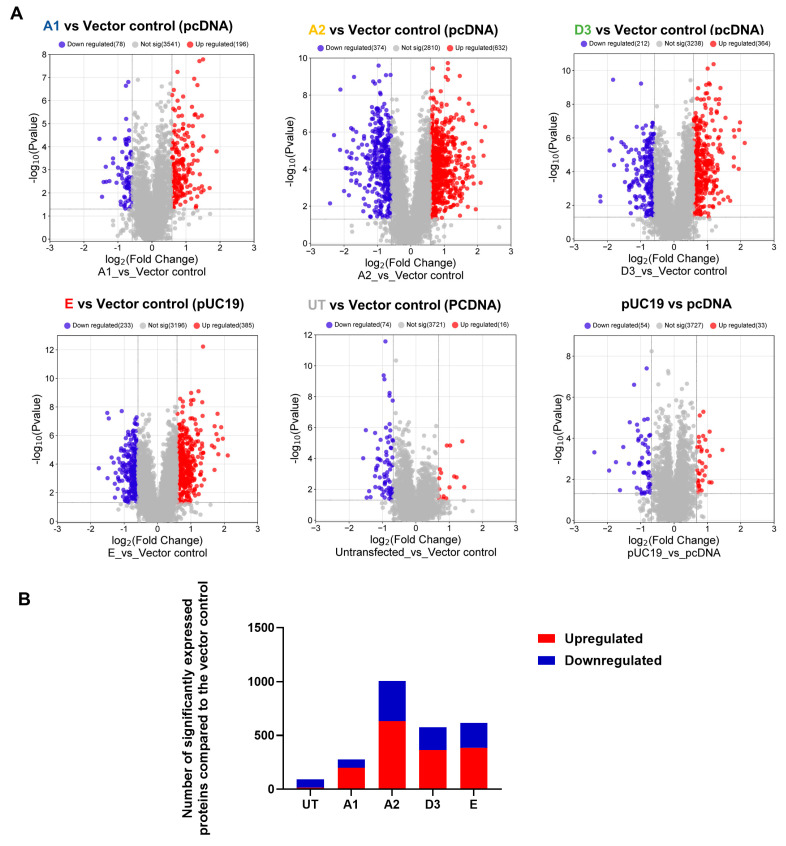
Mass spectrometry analysis of the HBV genotypes: (**A**) Volcano plots of the differentially expressed proteins in Huh7 cells transfected with replication-competent A1, A2, D3, E, and untransfected control against the vector-only control, using ©Spectronaut software. The negative *x*-axis represents downregulation (blue) in the vector control group, and the positive axis represents upregulated (red) proteins in the vector control group. (**B**) A bar graph showing the number of significantly upregulated (red) and downregulated (blue) proteins in each of the HBV (sub)genotypes, and the untransfected cells, compared to the vector control.

Volcano plots were generated to identify differentially expressed proteins in the transfected and untransfected cells (Figure 1A). Significantly differentially expressed proteins (DEPs) were classified into upregulated and downregulated proteins. A minimum fold change ≥1.5 and maximum false discovery rate (FDR)-adjusted *p*-value (q-value) of ≤0.05 were used to filter proteins that were significantly different between the various HBV (sub)genotypes and the vector control group. Firstly, the DEPs of the different HBV (sub)genotypes to the vector control were compared (Figure 1A). Compared to the vector control, the different (sub)genotypes showed different numbers of DEPs, which did not differ in the AVG log_2_ expression levels and range (Table 2). Subgenotype A1 had the least number of DEPs (323), whereas subgenotype A2 had the greatest number of DEPs (1025) (Figure 1B). However, subgenotype A1 showed the highest percentage of upregulated DEPs (72%) (Table 2). When comparing the commonly expressed proteins for all the (sub)genotypes, the F-box only protein (FBXO2) and Arginase type II (ARG2) were upregulated in subgenotype A1 compared to the remaining (sub)genotypes where they were downregulated. Compared to subgenotypes A2 and D3, the vitamin K epoxide reductase complex (VKORC1) and normal mucosa of oesophagus-specific gene-1 (NMES1) were upregulated in subgenotype A1. Splicing Factor-SWAP (SFSWAP) showed downregulation in subgenotype A1, while the other (sub)genotypes showed upregulation (Figure S3A,B).

**Figure 2:**
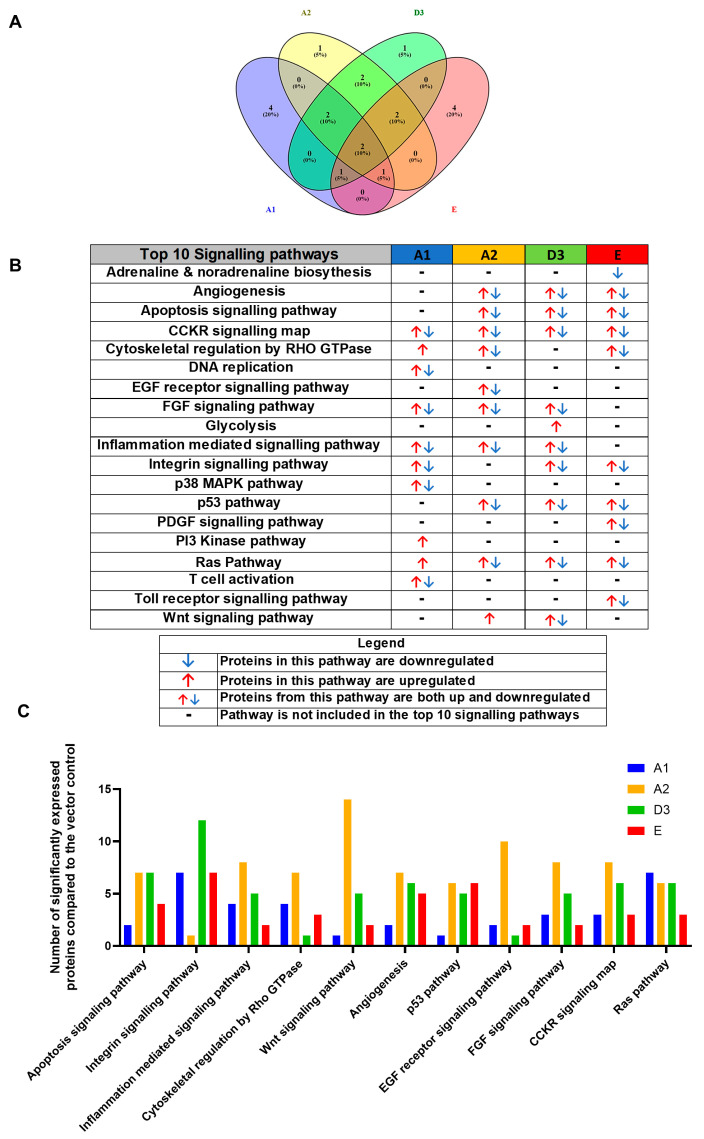
Proteomic analysis of the top 10 signalling pathways amongst the HBV genotypes. (**A**) Venn diagram indicating the pathways associated with differentially expressed proteins from A1, A2, D3, and E. Proteins were normalised against both the untransfected and vector-only control. (**B**) Comparison table demonstrating the top 10 pathways across the various genotypes. Proteomic analysis revealed significantly differently expressed proteins (*p* < 0.05) between the various (sub)genotypes These differentially expressed proteins were further classified into pathways. (**C**) Comparison table of the number of proteins amongst the top 10 pathways in (sub)genotypes A1, A2, D3, and E, which were present in more than one (sub)genotype.

For simplification, the DEPs were classified according to their corresponding pathways using the PANTHER bioinformatic tool, accessed 23 March 2023. The pathways were ranked according to the number of DEPs, and pathways with fewer than two proteins allocated were excluded from the analysis. When comparing the top 10 signalling pathways between (sub)genotypes A1, A2, D3, and E to the vector control, it was observed that the different (sub)genotypes favoured the dysregulation of specific host signalling pathways (Figure 2A). It should be noted that pathways marked with a (-) were not found in the top 10 signalling pathways but could still be dysregulated. The top 10 pathways shared between (sub)genotypes A1, A2, D3, and E when compared to the vector control were apoptosis, angiogenesis, cytoskeletal regulation by Rho GTPase, inflammation-mediated signalling, fibroblast growth factor (FGF) signalling, the wingless-related integration site (Wnt) signalling pathway, tumour p53 pathways, the rat sarcoma (RAS) pathway, the integrin signalling pathway, and the cholecystokinin receptor (CCKR) signalling pathway (Figure 2B). Interestingly, only the CCKR signalling and Ras pathways appeared in the top 10 categories amongst all the HBV genotypes. Subgenotype A1 caused dysregulation in seven proteins involved in the Ras signalling pathway (Figure 2C), whereas subgenotype A2 caused the most dysregulation in the Wnt pathway (13 DEPs), and subgenotype D3 caused the most dysregulation in the Integrin signalling pathway (12 DEPs). Interestingly, Genotype E did not favour a dysregulation of proteins in any of the top 10 signalling pathways (Figure 2C). The p53 pathway was not in the top 10 pathways dysregulated by subgenotype A1 relative to the vector control because only one protein was dysregulated in that pathway and the cut off used was >2 proteins. However, subgenotype A1 also showed dysregulation of a single protein in an alternate p53 pathway, the p53 pathway by glucose deprivation. No genes were dysregulated in the latter pathways by (sub)genotypes A2, D3, and E.

Out of the top 10 pathways dysregulated in the transfected cells, several were dysregulated only by a single subgenotype; exclusively by each of the different HBV (sub)genotypes as follows: T cell activation, PI3K pathway, DNA replication and P38 MAPK pathway by A1; EGF receptor signalling pathway by A2; glycolysis by D3; and PDGF signalling, adrenaline and noradrenaline receptor, and toll receptor signalling pathways by genotype E (Figure 2B). It should be noted that these pathways were exclusively found in the top 10 signalling pathways dysregulated by the different HBV (sub)genotypes, and this does not imply that these pathways were not dysregulated by other HBV (sub)genotype but were not in the 10 pathways dysregulated.

These results were confirmed using Gene Set Enrichment Analysis (GSEA), which is a computational method that determines whether a defined set of genes shows statistically significant, concordant differences between two biological states [47]. The proteome of Huh7 cells transfected with the various HBV (sub)genotypes was analysed by GSEA compared to the vector control. The significantly enriched genes were classified according to their oncogenic pathways. GSEA revealed the top exclusive oncogenic pathways for each (sub)genotype (Table 3). The hallmarks of cancer pathway PI3K_AKT_MTOR and epithelial_mesenchymal_ transition were significantly enriched for A1 and A2, respectively, whereas the glycolysis and IL2_STAT5_oncogenic pathways were enriched for subgenotype D3 and genotype E, respectively. Collectively, these results indicate that different HBV (sub)genotypes have a propensity to favour dysregulation in a specific host signalling pathway. No significant differences were observed in both the intracellular and extracellular viral load expression between the different (sub)genotypes 5 days post-transfection (Figure S2). However, differences in the expression of other viral proteins (e.g., HBsAg, HBeAg, etc.) which were not evaluated in this study may influence the proteome.

### 3.2. Hepatocarcinogenic Potential of Subgenotype A1 May Be Attributed to the Dysregulation of Ras-Associated Proteins

**Figure 3:**
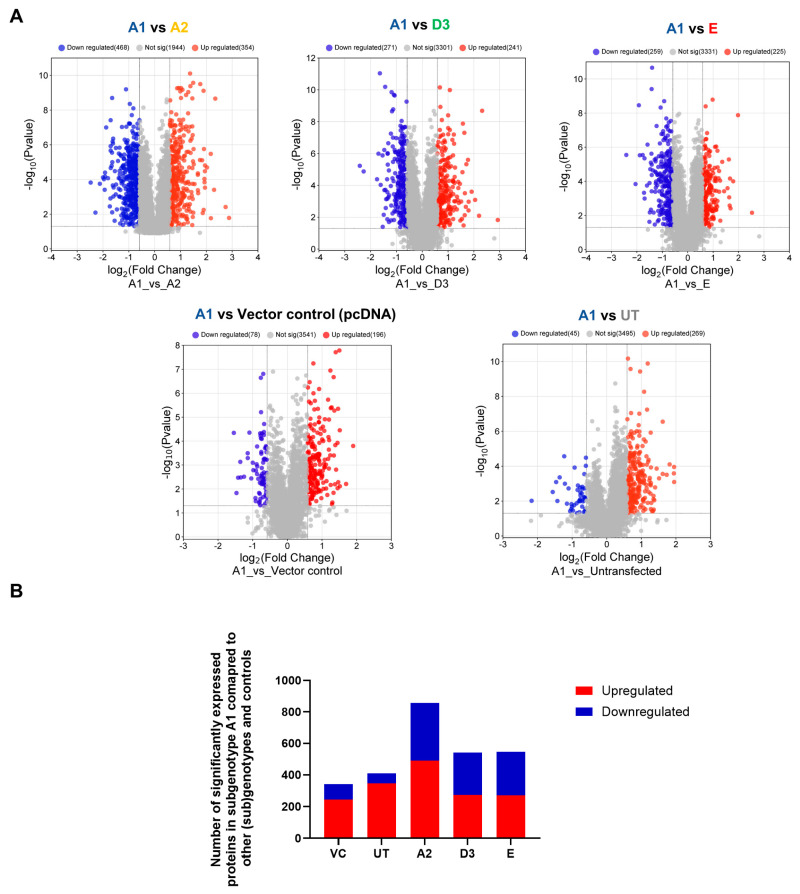
Mass spectrometry analysis of subgenotype A1 compared to different (sub)genotypes and controls. (**A**) Volcano plots of the differentially expressed proteins in Huh7 cells transfected with replication-competent A2, D3, E, and untransfected control, and vector control against subgenotype A1 using ©Spectronaut software. The negative *x*-axis represents downregulation (blue) in subgenotype A1, and the positive axis represents upregulated (red) proteins in subgenotype A1. (**B**) A bar graph showing the number of significantly upregulated (red) and downregulated (blue) proteins in each of the HBV (sub)genotypes, untransfected, and vector control, compared to subgenotype A1.

Previous studies have shown that subgenotype A1 has a higher hepatocarcinogenic potential compared to other (sub)genotypes [12]. The proteomic expression of the different (sub)genotypes was compared to determine whether different protein expressions may be responsible for differences that may contribute to the hepatocarcinogenic potential. Proteomic data suggest that subgenotype A1 favours the dysregulation of proteins involved in the Ras pathways compared to the other genotypes in the vector control background (Figure 2C). The dysregulated Ras proteins have been shown to affect downstream signalling in the P13K/mTOR/Akt signalling pathways, which was confirmed by the GSEA analysis between subgenotype A1 and the vector control (Table 3). The DIA SWATH–MS data analysis was repeated and used to compare quantifiable proteins in subgenotype A1 relative to the different HBV (sub)genotypes, using the same parameters as the previous analysis. A minimum fold change ≥1.5 and maximum FDR-adjusted *p*-value (q-value) ≤ 0.05 were used to filter proteins that were significantly different between subgenotype A1 and the various HBV (sub)genotypes (Figure 3A). Compared to subgenotype A2, subgenotype A1 showed the most DEPs (856), whereas subgenotype D3 (541) and genotype E (545) had a similar number of DEPs (Figure 3B). 

**Figure 4:**
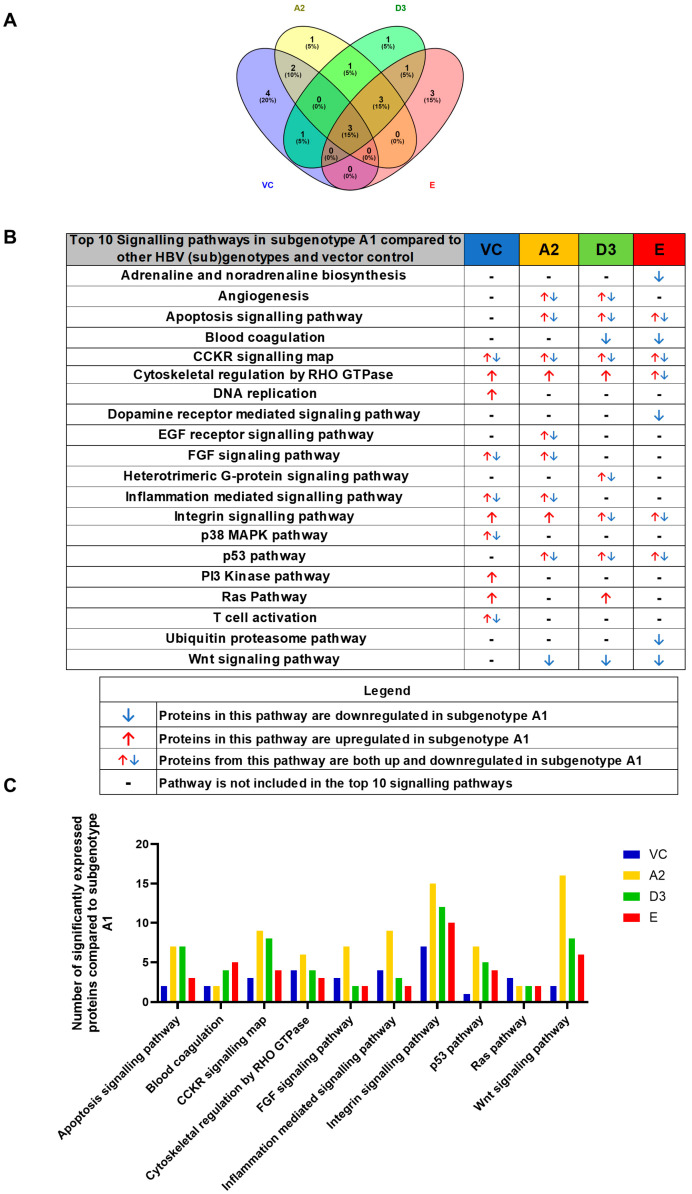
Proteomic analysis of the top 10 signalling pathways amongst the HBV genotypes: (**A**) Venn diagram indicating the pathways associated with differentially expressed proteins from A2, D3, E, and the vector control (VC). Proteomic analysis revealed significantly differently expressed proteins (*p* < 0.05) between the various (sub)genotypes These differentially expressed proteins were further classified into pathways. (**B**) Comparison table of the number of proteins amongst the top 10 pathways in subgenotype A1 compared to (sub)genotypes A2, D3, E, and the VC. (**C**) Comparison table of the number of proteins amongst the top 10 pathways in subgenotype A1 compared to (sub)genotypes A2, D3 and E, and vector control, which were present in more than one sample.

For simplification, the DEPs were classified according to their corresponding pathways using the PANTHER bioinformatic tool, accessed 23 March 2023. The pathways were ranked according to the number of DEPs, and pathways with fewer than two proteins allocated were excluded from the analysis. The top 10 signalling pathways present in subgenotypeA1 compared to the other HBV (sub)genotypes and the vector control were as follows: apoptosis, blood coagulation, CCKR signalling map pathway, cytoskeletal regulation by RHO GTPases, FGF signalling, inflammation-mediated signalling pathway, Integrin signalling pathway, p53 signalling pathway, Ras signalling pathway, and Wnt signalling pathway (Figure 4A). The CCKR signalling, cytoskeletal regulation by RHO GTPases, and integrin signalling pathways were dysregulated in A1 compared to each of the other (sub)genotypes and the vector control. Interestingly, only the CCKR signalling, RHO GTPases, and Integrin signalling pathways were common in the top 10 pathways involving subgenotype A1 compared to the other (sub)genotypes and vector control. With the exception of the blood coagulation pathway, subgenotype A2 had the highest number of DEPs in all of the pathways when compared to A1 (Figure 4B). Collectively, these results indicate that the greatest difference occurred between subgenotypes A1 and A2, revealing that even within the same genotype, the different subgenotypes can have varying effects on the Huh7 proteome.

**Figure 5:**
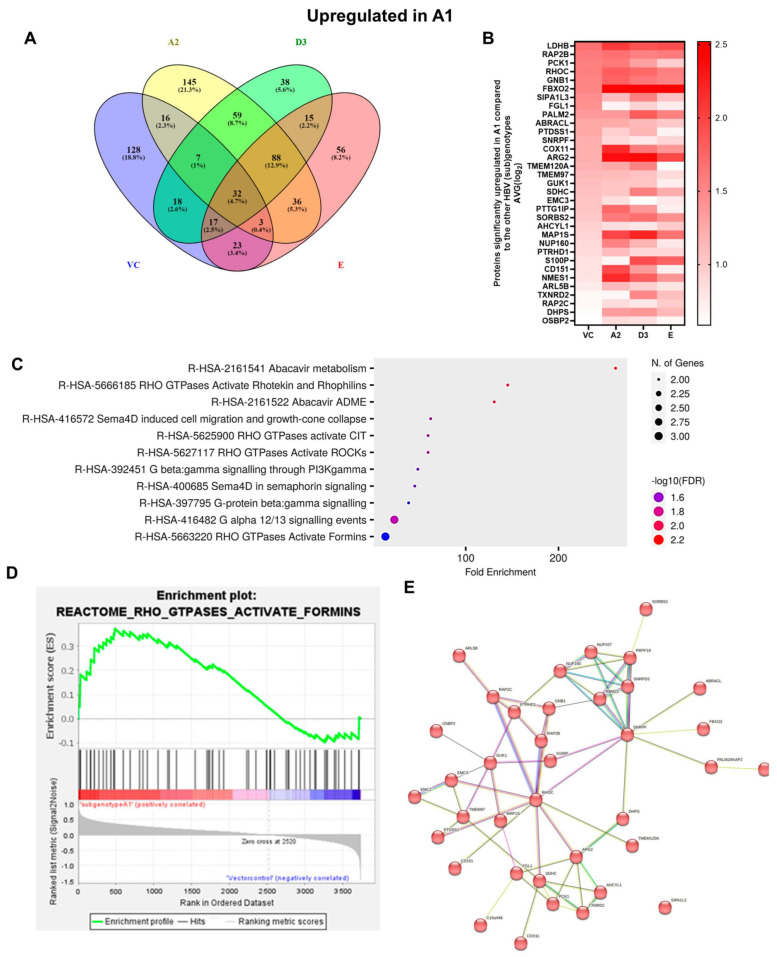
Upregulated common proteins found in subgenotype A1 in comparison to the other HBV (sub)genotypes. (**A**) Venn diagram illustrating the 32 significantly differentially expressed upregulated proteins found in A1 compared to the other HBV (sub)genotypes and the vector control. (**B**) The heatmaps show the upregulated (red) average log_2_ expression of the 32 significantly expressed common proteins in A1 compared to other HBV (sub)genotypes and the vector control. (**C**) A dot plot generated using the upregulated potential protein in ShinyGO analysis, with Reactome pathway enrichment and fold enrichment based on the number of genes present in each pathway. The FDR cut-off was set at 0.05, and the number of pathways was set to 10. (**D**) GSEA enrichment plot between subgenotype A1 and the vector control, showing the upregulated enrichment of Reactome pathway of RHO GTPases activating formins. (**E**) Network analysis of upregulated dysregulated proteins using STRING network (Fold cut-off set to 0.4). Lines of different colors represent seven types of evidence used in predicting associations. Red line: fusion evidence; green line: neighborhood evidence; blue line: co-occurrence evidence; purple line: experimental evidence; yellow line: text mining evidence; light blue line: database evidence; black line: co-expression evidence.

**Figure 6:**
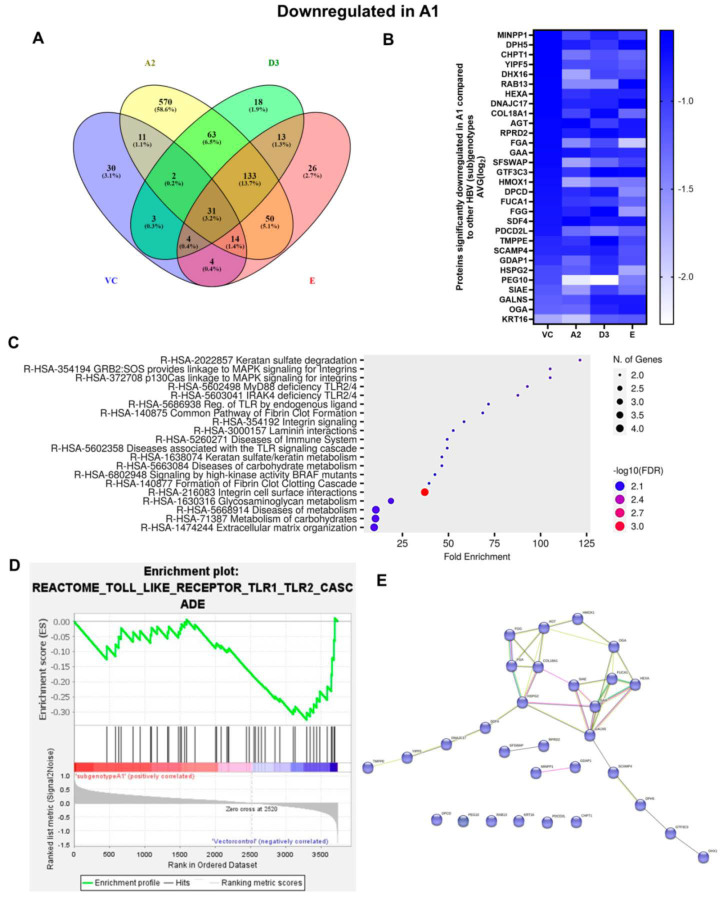
Downregulated common proteins found in subgenotype A1 in comparison to the other HBV (sub)genotypes. (**A**) Venn diagram illustrating the 31 significantly differentially expressed downregulated proteins found in A1 compared to the other HBV (sub)genotypes and the vector control. (**B**) The heatmaps show the downregulated (blue) average log_2_ expression of the 32 significantly expressed common proteins in A1 compared to other HBV (sub)genotypes and the vector control. (**C**) A dot plot generated using the downregulated potential protein in ShinyGO analysis, with Reactome pathway enrichment and fold enrichment based on the number of genes present in each pathway. The FDR cut-off was set at 0.05, and the number of pathways was set to 10. (**D**) GSEA enrichment plot between subgenotype A1 and the vector control, showing the downregulated enrichment of the Reactome pathway of Toll-like receptor TLR1–TLR2 cascade. (**E**) Network analysis of downregulated dysregulated proteins using STRING network (Fold cut-off set to 0.4). Lines of different colors represent seven types of evidence used in predicting associations. Red line: fusion evidence; green line: neighborhood evidence; blue line: co-occurrence evidence; purple line: experimental evidence; yellow line: text mining evidence; light blue line: database evidence; black line: co-expression evidence.

Next, common upregulated (32) and downregulated (31) DEPs in subgenotype A1 compared to the various HBV (sub)genotypes were identified, as shown in Figure 5 and Figure 6, respectively. ShinyGO Gene Ontology enrichment analysis [48] was performed using the differentially expressed common up- and downregulated proteins in subgenotype A1 compared to the other HBV (sub)genotypes and vector control. The average (AVG) log_2_ expression in A1 relative to vector control and other genotypes is represented in Table 4 below. 

FBOX2 was the only protein upregulated in subgenotype A1, relative to A2, D3 and E, at an AVG log_2_ expression > 2, whereas ARG2 was upregulated at an AVG log_2_ expression > 2, relative to A2 and D3. Three proteins, lactase dehydrogenase B (LDBH), cytochrome c oxidase copper chaperone (COX11), and NMES1, were upregulated at an AVG log_2_ expression > 2, only relative to A2.

The enrichment of 11 similar upregulated pathways (Figure 5C) and 20 similar downregulated pathways was observed (Figure 6C). Furthermore, the data confirmed the enrichment of activated pathways through several interconnecting networks using STRING analysis (Figure 5E and Figure 6E). The common upregulated proteins were involved in cell proliferation, apoptotic resistance, evasion, and metastasis processes (Figure 5C). These included the following Reactome pathways: RHO GTPases activating formins, G alpha 12/13 signalling events, G Beta—Gamma signalling through PI3K signalling, RHO GTPases activating ROCKS, and G protein gamma signalling pathways, etc. The common downregulated proteins were involved in immunosuppression and carbohydrate metabolism (Figure 6C). These included the following Reactome pathways: Extracellular matrix organisation, metabolism of carbohydrates, disease of metabolism, disease of immune system and disease associated with the TLR signalling cascade, etc. and others. 

Amongst the 32 commonly upregulated proteins, 3 proteins of interest were identified, namely guanine nucleotide-binding protein G(I)/G(S)/G(T) subunit beta-1 (GNB1), Rho-related GTP-binding protein (RHOC), and Ras-Associated Protein 2b (Rap2b), for their involvement in the Ras pathway (Figure 5). Each of these proteins was significantly upregulated (>1.5 fold change increase) in subgenotype A1 compared to other (sub)genotypes and the vector control (Figure 5B).

**Figure 7:**
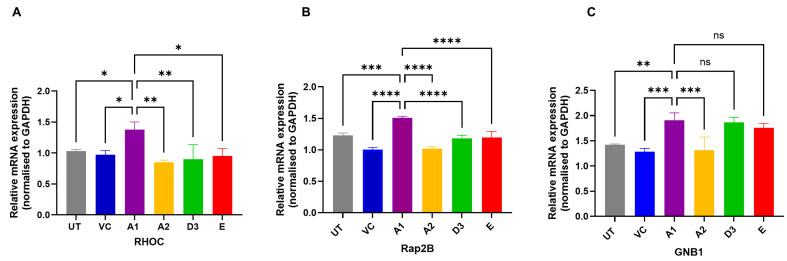
Verification of the potential Ras-associated proteins. Ras-associated proteins RHOC (**A**), Rap2B (**B**), and GNB1 (**C**) were verified as potential proteins by measuring the mRNA expression normalised to the GADPH housekeeping control 5 days post-transfection. Results are expressed as a ratio between the mRNA expression of the Ras-associated proteins and GAPDH. Significant differences were analysed using a one-way ANOVA statistical test and compared to the expression in subgenotype A1. * = *p*-value < 0.05, ** = *p*-value < 0.01, *** = *p*-value < 0.001, **** = *p*-value < 0.0001 and ns = not significant.

The increased expression of the Ras-associated proteins in subgenotype A1 compared to other HBV (sub)genotypes was verified on an RNA level, using RT-qPCR. mRNA expression levels of the Ras-associated proteins 5 days post-transfection revealed that RHOC and Rap2B were significantly upregulated in A1 compared to the other HBV genotypes, vector control, and the untransfected control, validating the results from the SWATH–MS (Figure 7A,C) using a one-way ANOVA statistical test. However, mRNA expression levels of the Ras-associated protein GNB1 only revealed a significant increase in subgenotype A1 compared to subgenotype A2, the vector control, and the untransfected control, using a one-way ANOVA statistical test (Figure 7C).

### 3.3. Subgenotype A1 Influence on RHOC and Its Downstream Signalling Products

**Figure 8:**
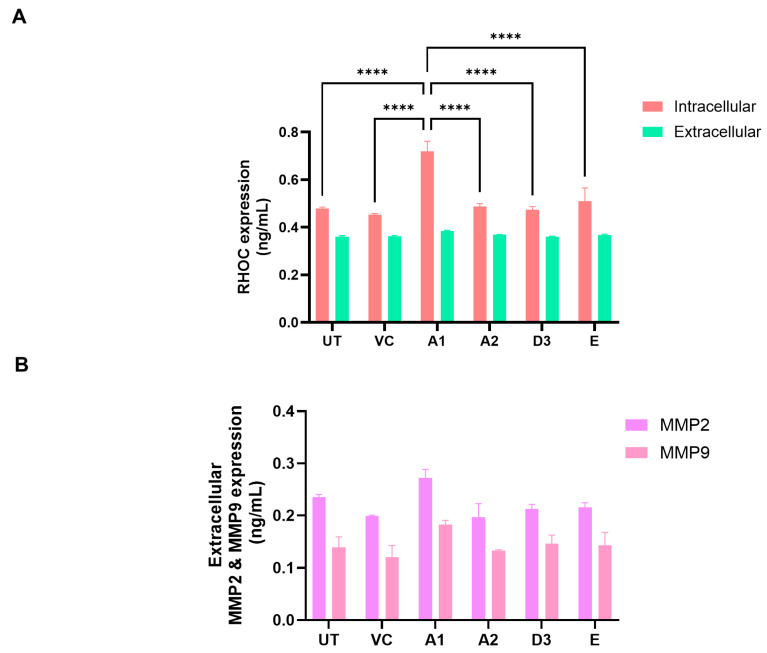
The expression of RHOC and its downstream signalling products. (**A**) Intracellular (red) and extracellular (green) expression of RHOC in Huh7 cells transfected with the various HBV (sub)genotypes after 5 days. Significant differences were analysed using a one-way ANOVA statistical test and compared to the expression in subgenotype A1 (**** = *p*-value < 0.0001). (**B**) The extracellular supernatant was harvested from HBV (sub)genotypes and controls, 5 days post-transfection, and analysed with MMP2 and MMP9 ELISA kit. Results indicated that MMP2 (purple) extracellular expression levels were significantly higher than MMP9 expression levels (pink) using a two-way ANOVA statistical test. Subgenotype A1 showed a non-significant increase in both MMP2 and MMP9 expression levels compared to the other HBV (sub)genotypes and controls.

Gene enrichment analysis using both ShinyGO 8.0 and GSEA between subgenotype A1 and the vector control (Figure 5C,D) indicated that the top common pathway amongst the 32 upregulated proteins in subgenotype A1 compared to the other HBV (sub)genotypes and vector control was RHO GTPases activating formins using the Reactome pathways. Recently, mounting evidence has demonstrated that the Rho GTPase family plays an important role in mediating HCC progression [49]. Therefore, overexpression of RHOC was explored as a protein of interest for subgenotype A1. The overexpression of RHOC in subgenotype A1, compared to the other HBV (sub)genotypes and vector control was further verified on a proteomic level by ELISA. We examined both the intercellular and extracellular expression of RHOC 5 days post transfection and found that intracellular RHOC expression was significantly higher in subgenotype A1 compared to other HBV (sub)genotypes and controls (untransfected and vector control), validating the results from the DIA SWATH-MS using a one-way ANOVA statistical test (Figure 8A). These results agreed with mass spectrometry and mRNA expression results. However, as expected, there was no difference in the extracellular expression of RHOC, as it is only expressed intracellularly [50].

Next, downstream extracellular signalling products activated by RHOC overexpression matrix metalloproteinase 2 (MMP2) and matrix metalloproteinase 9 (MMP9) were tested. The extracellular MMP2 and MMP9 ELISA revealed a non-significant increase in MMP2 and MMP9 in the supernatant of the subgenotype A1-transfected cells compared to those transfected with the other (sub)genotypes 5 days post-transfection (Figure 8B).

**Figure 9:**
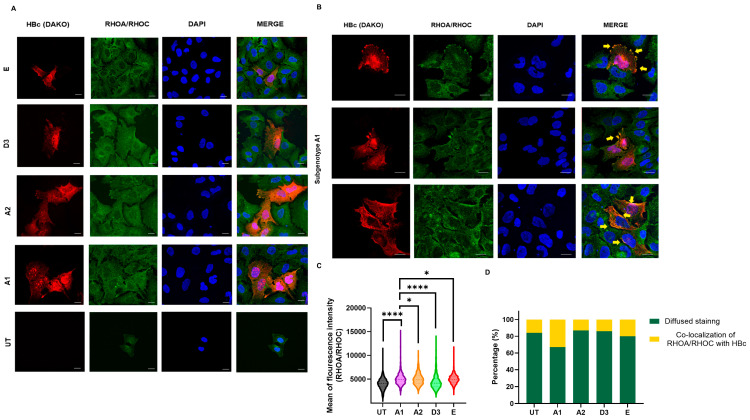
The localisation of RHOC expression in the various (sub)genotypes. (**A**) Microscopy of transfected Huh7 cells with the different HBV (sub)genotypes after 5 days, viewed with a fluorescent microscope. Cells were immunostained with a polyclonal rabbit anti-HBc antibody (DAKO) and monoclonal mouse anti-RHOA/RHOC antibody. The nuclei were visualised by DAPI staining. (**B**) Selected microscopy of transfected Huh7 cells with subgenotype A1 after 5 days, viewed with a fluorescent microscope. Cells were immunostained with a polyclonal rabbit anti-HBc antibody (DAKO) and monoclonal mouse anti-RHOA/RHOC antibody. The nuclei were visualised by DAPI staining. Yellow arrows indicate the areas of focal adhesion where RHOA/RHOC is co-localised with the HBV core protein. (**C**) A volcano plot indicating the mean fluorescence intensity of the RHOA/RHOA expression in cells transfected with subgenotype A1 compared to other HBV (sub)genotypes and the untransfected (UT) control. Significant differences were analysed using a one-way ANOVA statistical test and compared to the expression in subgenotype A1. * = *p*-value < 0.05 and **** = *p*-value < 0.0001. (**D**) Comparative bar chart showing the percentage of Huh7 cells that have displayed diffused RHOA/RHOC staining or focal adhesion staining, amongst the various HBV genotypes.

Using the RHOA/RHOC antibody, mean fluorescent intensity was significantly higher in the subgenotype A1-transfected cells compared to the other (sub)genotypes and the untransfected control (Figure 9A). The majority of Huh7 cells transfected with the HBV (sub)genotypes displayed a diffused gradient staining of the cytoplasm throughout the cell. Co-localisation of the RHOA/RHOC antibody and the HBV core protein (HBc) was observed in 35% of the Huh7 cells transfected with subgenotype A1, compared to the other HBV genotypes and untransfected control, which showed a lower range of 15–20% (Figure 9B,C). This localisation occurred mainly at the periphery of the cells, intimating the role of RHOC as a regulator of focal cell adhesion and in lobopodial migration [51]. It should be noted the anti-HBc (Dako), which is used to detect viral proteins in transfected cells, does not differentiate between core and precore proteins. 

## 4. Discussion

HBV has a significant pathogenic potential, which is associated with both the ability to establish chronic infection, persist, and ultimately lead to the clinical manifestation of cirrhosis and/or HCC [52]. The course and outcomes of the infection depend on several virus–host interactions [53]. Although the incidence of chronic HBV infection (CHB) is declining globally, its incidence and associated HCC morbidity and mortality has increased in SSA [3,54]. Genetic variation in the HBV prevailing in SSA may play an important role in the virologic and clinical characteristics of infection, impacting disease progression and the response to antiviral therapy [20].

Using a DIA SWATH–MS-based quantitative proteomic approach, a comparative analysis of the whole proteome of Huh7 cells transfected with the different SSA HBV (sub)genotypes relative to subgenotype A2 was performed. Huh7 cells were used instead of HepG2 cells because the former are less sensitive to stress and serum deprivation [55]. To the best of our knowledge, this study represents the first of its kind. The different SSA HBV (sub)genotypes, compared to the vector control (Figure 2) or to each other (Figure 4), dysregulated specific host signalling pathways. For the generation of the plasmids, the sequence closest to the consensus sequence of each (sub)genotype was used to ensure the representation of the (sub)genotypes [39]. Although subgenotypes A1 and A2 belong to the same genotype, they demonstrate a higher divergence in the BCP/precore region between each other than between subgenotype A1 and (sub)genotypes D3 and E, respectively [20]. This could be the reason why the greatest difference in the proteome was between two subgenotypes of A, A1 and A2, and not between two different genotypes. Even though subgenotype A2 had the highest number of DEPs compared to subgenotype A1, the latter had a higher percentage of upregulated DEPs (Table 2). 

Subgenotypes A1 and A2 have different clinical manifestations [11,20,27]. Subgenotype A1 develops different mutations in the BCP and pre-core region, whereas subgenotype A2 only develops mutations in the BCP, decreasing precore mRNA levels, which affect the expression level of HBeAg [11,20,27], resulting in significantly higher HBeAg negativity in subgenotype A1-infected patients than subgenotype A2-infected patients [21,22]. Accumulation of viral proteins such as HBeAg and HBsAg can lead to ER stress [31,56,57]. An in vitro study showed that Huh7 cells transfected with subgenotype A1 had increased ER stress and higher PERK, ATF6, and IRE1/XBP1 activity than cells transfected with subgenotype A2 and D3 [31]. In addition, FBXO2, which is an ubiquitin ligase, was the only protein upregulated in subgenotype A1 relative to A2, D3, and E, at an AVG log_2_ expression >2 (Figure 5B). The persistent activation of the unfolded protein response (UPR) when the cells were transfected with subgenotype A1 may explain why even though it had the least number of DEPs, it still had a higher hepatocarcinogenic potential. Additional research will be beneficial in elucidating this paradox.

### 4.1. Dysregulated Signalling Pathways 

Four pathways, p53, ERK/MAPK, Wnt/β-catenin, and PI3K/Akt/mTOR were identified as part of the top 10 signalling pathways across the different (sub)genotypes when compared to the vector control (Figure 2) and were also dysregulated in subgenotype A1 when compared to the other HBV (sub)genotypes and vector control (Figure 4). Previous studies on the mechanism of HBV-induced HCCs have identified five main signalling pathways which are dysregulated in the host as follows: the p53, oxidative stress, ERK/MAPK, PI3K/Akt, and Wnt/β-catenin signalling pathways [34,37,52,58]. The oxidative stress pathway was the only pathway not in the top 10 dysregulated pathways identified in this study (Figure 2 and Figure 4).

#### 4.1.1. p53 Pathway

The p53 signalling pathway was dysregulated in subgenotype A1 compared to the different HBV (sub)genotypes but not relative to the vector control, as only one protein was dysregulated in vector control and the cut-off used was >2 proteins. However, subgenotype A1 also showed dysregulation of a single protein in an alternate p53 pathway, the p53 pathway by glucose deprivation. The p53 by glucose deprivation pathway is activated in ER stress [59], which is increased in subgenotype A1 compared to the other (sub)genotypes [31]. It is possible that subgenotype A1 dysregulates p53 by alternative pathways compared to the other subgenotypes. Proteins involved in the p53 pathway were both upregulated and downregulated in subgenotype A1 compared to the other HBV (sub)genotypes (Figure 4A,B). This could be the result of the multiple feedback loops associated with this signalling pathway [60]. Mutations in the p53 tumour suppressor gene are found in 12–48% HBV-associated HCC cases [58,61,62,63]. These mutations result in the inactivation of p53 and cell cycle pathway activation and they are associated with tumour aggressiveness and poor prognosis [64]. It would be interesting to determine if these mutations occur more frequently in HCC patients infected with subgenotype A1. 

#### 4.1.2. MAPK Pathway

The p38 MAPK signalling pathway was identified as one of the top signalling pathways dysregulated between cells transfected with subgenotype A1 and the vector control. Furthermore, gene enrichment analysis between the common downregulated proteins in subgenotype A1 compared to the other HBV (sub)genotypes or vector control showed enrichment in the immune system pathways (Figure 6C,D), thereby allowing for the persistence of subgenotype A1. MAPK signalling has been shown to play an important role in immune escape as well as HBV replication, the promotion of tumour cell survival and drug resistance [52]. In fact, p38 MAPK has been demonstrated to play a crucial role in HBsAg/HBeAg secretion, viral replication, and the formation of HBV cccDNA in HBV-infected cells [65,66]. This downregulation in MAPK would allow subgenotype A1 to evade immune surveillance and thus persist. In SSA, subgenotype A1 is transmitted before the age of one year [67,68], when 90% of infections subsequently lead to chronic infection and can be associated with the early development of HCCs [22]. 

The coagulation cascade signalling pathway has been shown to mediate both host innate and adaptive immunity against viral infection [69]. As one of the top ten pathways, the blood coagulation pathway was downregulated in Huh7 cells transfected with subgenotype A1, but not in cells transfected with either of the (sub)genotypes of D3 or E (Figure 4). Dysregulation of the complement cascade was also demonstrated following proteomic analyses of the plasma derived from patients infected with either genotypes B or C [38]. Thus, the downregulation in the coagulation signalling pathway may be an additional mechanism for immune evasion by subgenotype A1, which requires further investigation.

#### 4.1.3. Wnt/β-Catenin Pathway

The Wnt signalling pathway was downregulated in subgenotype A1 compared to the other HBV (sub)genotypes. On the other hand, subgenotype A2 showed an upregulation in 13 DEPs, leading to the dysregulation of the Wnt signalling pathways (Figure 2C). An abnormal activation of the Wnt/β-catenin signalling pathway has been observed in approximately 66% of patients with HCC, and the activation of this pathway is closely related to the occurrence, development, stemness, and drug resistance of HCC [70,71,72]. Similarly, the CTNNB1 mutation found in HCC tumour cells but not in adjacent non-tumour cells has been demonstrated to activate the Wnt pathway [34].

Additionally, the presence of viral proteins HBx and HBsAg drives Wnt signalling in HBV-infected patients. HBx dysregulates the Wnt pathway through several mechanisms, including the regulation of GSK3β, β-catenin, Frizzled receptor, and URG7 [72]. Considering that the HBx of subgenotype A1 differs from that of other (sub)genotypes [73] and its effect on the Wnt pathway has not been studied, the effect of A1 HBx on Wnt may also be different. Furthermore, HBsAg modulates the expression of lymphoid enhancer-binding factor 1(LEF-1), a downstream mediator and transcription factor of the Wnt signalling pathway [74]. Studies have shown that A2 has the highest intracellular expression levels of HBsAg compared to subgenotypes A1, B1, D1, C1, and F1b [75,76]. This increase in intracellular HBsAg could explain why subgenotype A2 favours dysregulation of the Wnt signalling pathway (13 DEPs).

#### 4.1.4. PI3K/Akt/mTOR Pathway

Proteomic analysis using GSEA revealed that the top dysregulated pathway was PI3K/Akt/mTOR pathway (Table 1). Furthermore, gene enrichment analysis between the common upregulated proteins in cells transfected with subgenotype A1 compared to the other HBV (sub)genotypes or vector control showed enrichment in genes, which were upregulated in the PI3K signalling (Figure 5). The PI3K/Akt/mTOR pathway is overexpressed in nearly 50% of HCCs, and the dysregulated activation of this pathway affects a wide range of processes, including cell proliferation, metabolism, tumour cell differentiation, lipid metabolism, autophagy, and epithelial to mesenchymal transition (EMT) [77,78]. Furthermore, previous studies have shown that the interaction of the mTOR signalling pathway and HBV life cycle is complex. The mTOR signalling pathway can regulate HBV replication and gene expression [79,80,81], whereas HBV infection can activate the PI3K/Akt/mTOR pathway. This occurs following the synthesis of a large quantity of viral proteins, which leads to the accumulation of HBsAg/HBeAg in the endoplasmic reticulum (ER) lumen that then triggers ER stress [31,82]. A previous study using the same plasmid construct showed that subgenotype A1 exhibited a greater amount of ER stress compared to subgenotypes A2 and D3 [31], which could possibly lead to the activation of the PI3K/Akt/mTOR pathway. 

### 4.2. Dysregulated Proteins 

**Figure 10:**
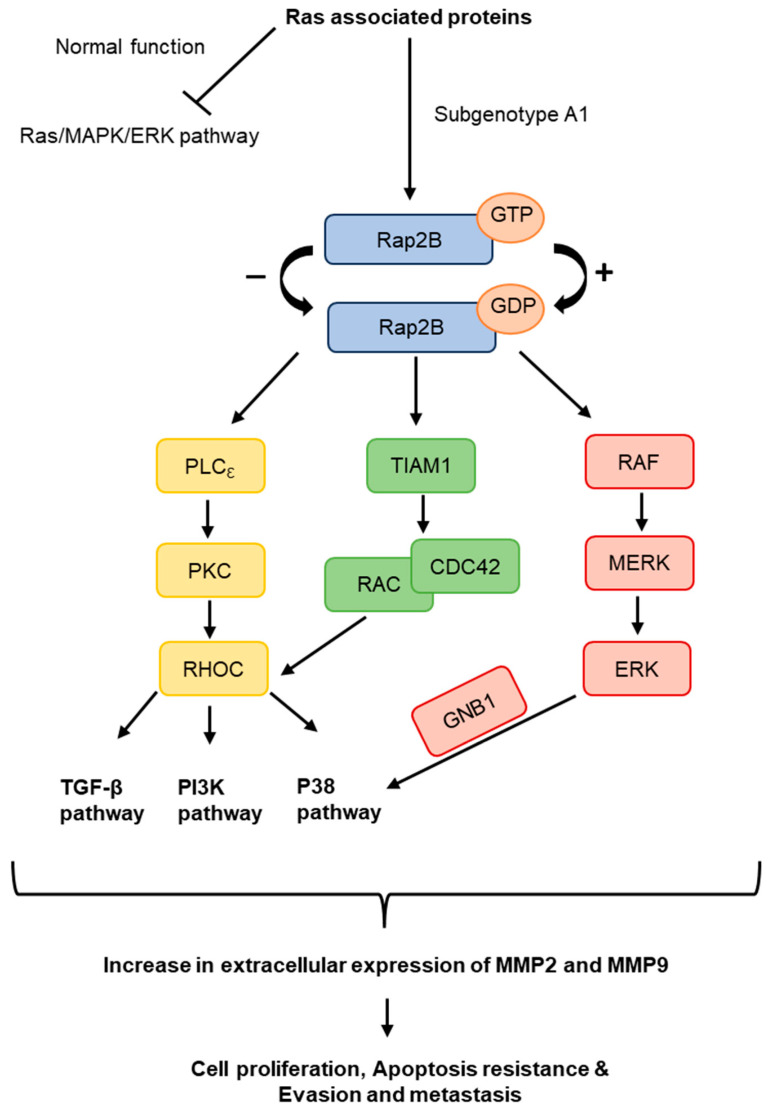
The role of potential Ras-associated proteins upregulated in A1. The predicted overexpressed or activated signalling Ras pathway that subgenotype A1 favours. Potential Ras-associated proteins, Rap2B, RhoC and GNB1, are seen throughout the pathway and can affect downstream signalling pathways, TGF-β, PI3K/Akt, and p38 signalling pathways. These downstream signalling pathways have been shown to increase cell proliferation, apoptosis resistance, evasion, and metastasis in HCCs by increasing extracellular MMP2 and MMP9 expression.

Further analysis was performed to identify proteins that were commonly upregulated and affected in more than one of the four dysregulated pathways in the cells transfected with subgenotype A1, compared to the other (sub)genotypes or vector control. Three significantly upregulated Ras-associated proteins were identified. These included Ras homolog gene family, member C (RHOC), Ras-related protein-2b (Rap2B), and GNB1, which are upstream regulators of the MAPK and PI3K/Akt/mTOR pathways at the protein level (Figure 10). Both the MAPK and PI3K signalling pathways were also exclusively found in the top 10 signalling pathways with dysregulation from subgenotype A1 when compared to the vector control (Figure 2B), whilst the PI3K pathway was found to be the top exclusive oncogenic pathway in subgenotype A1 compared to the vector control using GSEA analysis (Table 3). These pathways regulate cell growth, motility, survival, and metabolism [83,84,85,86] (Figure 10). 

Quantitative reverse transcription PCR was performed to confirm this upregulation. The mRNA levels of RHOC and Rap2B were significantly upregulated in subgenotype A1 relative to the other HBV (sub)genotypes or vector control (Figure 7A,B), whereas GNB1 mRNA expression was not significantly upregulated relative to D3 and E (Figure 7C), which could be a result of the post-translation steps required for the maturation and expression of GNB1 [87,88].

Proteomic analyses in the cells transfected with subgenotype A1 compared to the different HBV (sub)genotypes or vector control showed enrichment in multiple pathways where RHO GTPases activity is upregulated (Figure 5C,D). Thus, RHOC expression was further analysed. The significant upregulation in RHOC in the subgenotype A1-transfected cells was confirmed using a RHOC-specific ELISA, as a secondary proteomic assay (Figure 7B). 

Next, because RHOC is an important regulator of cell migration [89] and involved in cancer progression, especially metastasis in various cancers including HCC [49,89], the cellular localisation of RHOC in the Huh7 cells transfected with subgenotype A1 was compared to the other (sub)genotypes or untransfected cells. Furthermore, overexpression of RHOC has been implicated in poor clinical prognosis because of its association with metastatic and aggressive features of HCC [49]. To avoid cross-reactivity between the primary antibodies used to detect the RHOC and the Dako (anti-HBc), we used a combination RHOA/RHOC antibody. RHOA and RHOC are both located in the cytoplasm; however, their functionality differs [90]. RHOA plays a key role in regulating actomyosin contractility and cell proliferation, whereas RHOC is more important in cell movement [91]. In cells transfected with subgenotype A1, increased co-localisation of RHOA/RHOC and HBc was observed at the periphery of a majority of the cells (Figure 9). This observation agrees with a study by Xie et al. which showed that the stable transfection of the RHOC expression vector into a normal hepatocyte cell line imparted tumour phenotypes, including proliferation, anchorage-independent growth, migration, and invasion [92]. The increased focal adhesion staining of RHO proteins in the subgenotype A1-transfected cells compared to cells transfected with other (sub)genotypes may be related to higher hepatocarcinogenic potential of this subgenotype. More in-depth studies are necessary. 

Downstream signalling products of RHOC, MMP2, and MMP9 have been shown to be increased [93,94]. Therefore, extracellular MMP2 and MMP9 levels were compared using ELISA. Although not statistically significant, the increased extracellular expression of MMP2 and MMP9 was detected 5 days post-transfection in the supernatant of the subgenotype A1-transfected cells compared to those transfected with the other (sub)genotypes (Figure 8B). RHOC has been shown to promote the malignant progression of cholangiocellular carcinoma cells via the regulation of MMP 2, 3, and 9 expression levels [95,96]. Moreover, Liao et al. reported that HCC cell invasion and migration are modulated by the genes RHOC, MMP2, and MMP9 [97].

## 5. Conclusions

There were a number of limitations present in this study, including the use of Huh7 cells derived from a hepatoma, the lack of a secondary proteomic assay for GNB1, and the use of RHOA/RHOC antibody instead of a RHOC-specific antibody. Despite these limitations, using proteomic analysis, subgenotype A1 was demonstrated to dysregulate four of the five HBV-associated hepatocarcinogenic pathways—p53, MAPK, Wnt/β-catenin, and PI3K/Akt/mTOR. The upstream regulator proteins (RHOC, Rap2B, and GNB1) of the MAPK and PI3K/Akt/mTOR pathways were also shown to be upregulated in the cells transfected with subgenotype A1. Furthermore, the increased expression and localisation of RHO proteins in the subgenotype A1-transfected cells compared to cells transfected with other (sub)genotypes may be related to the higher hepatocarcinogenic potential of this subgenotype. As an in vitro system, transfection of the Huh7 cells with HBV plasmid constructs provides us with a widely used model system that allows us to follow HBV infection and dissect complex biological processes [98]. However, to further elucidate the mechanisms of the pathogenesis of subgenotype A1-associated HCCs, further in vivo studies using patient tissues and/or sera will be required. 

## Figures and Tables

**Table 1:** Primers use for reverse transcriptase qPCR.

Gene	Direction	Sequence
RHOC	F	5′-TCCTCATCGTCTTCAGCAAG-3
	R	5′-CTGCAATCCGAAAGAAGCTG-3′
Rap2B	F	5′-TTACCGCAAGGAGATTGAG-3′
	R	5′-GGCTGTAGACCAGGATGAAG-3′
GNB1	F	5′-TGCTTGGAGAGTGTGGGCTTCT-3′
	R	5′-TGGAGGGCATCTCCAGAATCTG-3′
GAPDH	F	5′-GAAGGTGAAGGTCGGAGT-3′
	R	5′-GAAGATGGTGATGGGATTTC-3′

**Table 2:** AVG log_2_ expression of differentially expressed proteins found in the different HBV (sub)genotypes A1 compared to the vector control.

Expression	(Sub)genotype	Mean AVG log_2_ Expression	AVG log_2_ Range	Mean *p*-Value	% of DEPs
**Upregulated**	A1	0.90	0.58–1.98	0.0073764	72
	A2	0.91	0.58–2.26	0.0027466	63
	D3	0.85	0.58–2.11	0.0032396	63
	E	0.87	0.58–2.71	0.0030094	62
**Downregulated**	A1	−0.89	−0.58–−2.18	0.0150298	28
	A2	−0.92	−0.58–−2.30	0.0040270	37
	D3	−0.84	−0.58–−2.21	0.0070993	37
	E	−0.86	−0.58–−2.45	0.0099477	38

**Table 3:** GSEA analysis of the top exclusive oncogenic pathway and the associated proteins dysregulated in Huh7 cells amongst the different HBV (sub)genotypes.

(Sub)genotypes	Top Exclusive Dysregulated Oncogenic Pathway Compared to the Vector Control	
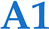	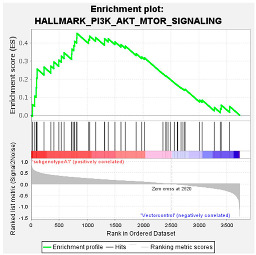	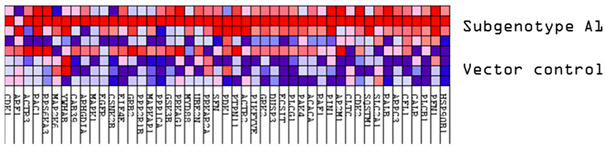
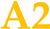	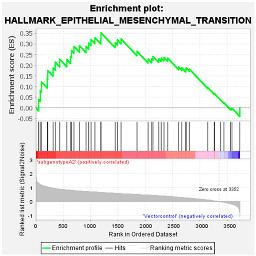	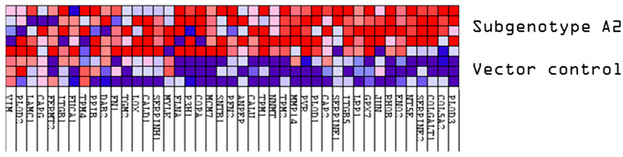
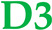	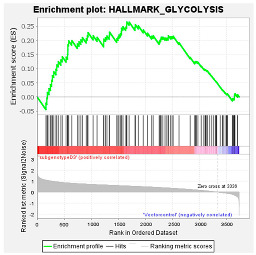	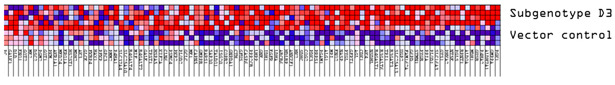
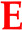	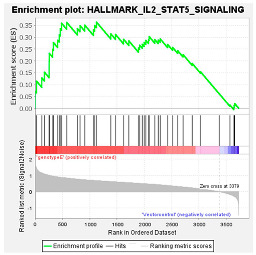	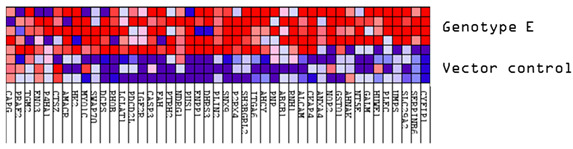

**Table 4:** AVG log_2_ expression of the common differentially expressed proteins found subgenotypes A1 compared to the other (sub)genotypes.

Expression	(Sub)genotype/Control	Mean AVG log_2_ Expression	Mean AVG log_2_ Range	Mean *p*-Value
**Upregulated**	VC	1.10	0.59–1.66	0.0074896
	A2	1.40	0.64–2.92	0.0038629
	D3	1.40	0.61–2.45	0.0045624
	E	1.73	0.60–2.77	0.0083334
**Downregulated**	VC	−0.82	−0.60–−1.62	0.0159729
	A2	−1.18	−0.59–−2.12	0.0044853
	D3	−1.00	−0.61–−2.26	0.0042862
	E	−1.11	−0.60–−1.92	0.0066141

## Data Availability

The raw data supporting the conclusions of this article will be made available by the authors upon request.

## Supplementary Materials

The following supporting information can be downloaded at: https://www.mdpi.com/article/10.3390/v16071052/s1, Figure S1: Internal validation of mass spectrometry data; Figure S2: Intracellular and extracellular viral load expression of the various HBV (sub)genotypes; Figure S3: Commonly expressed protein in HBV (sub)genotypes compared to the vector control.
