# Supplementary figures and images for: Comparative Proteomic Analysis of Huh7 Cells Transfected with Sub-Saharan African Hepatitis B Virus (Sub)genotypes Reveals Potential Oncogenic Factors
Source: Viruses. 2024 Jun 29;16(7):1052. doi: 10.3390/v16071052 (PMC11281506; doi:10.3390/v16071052)

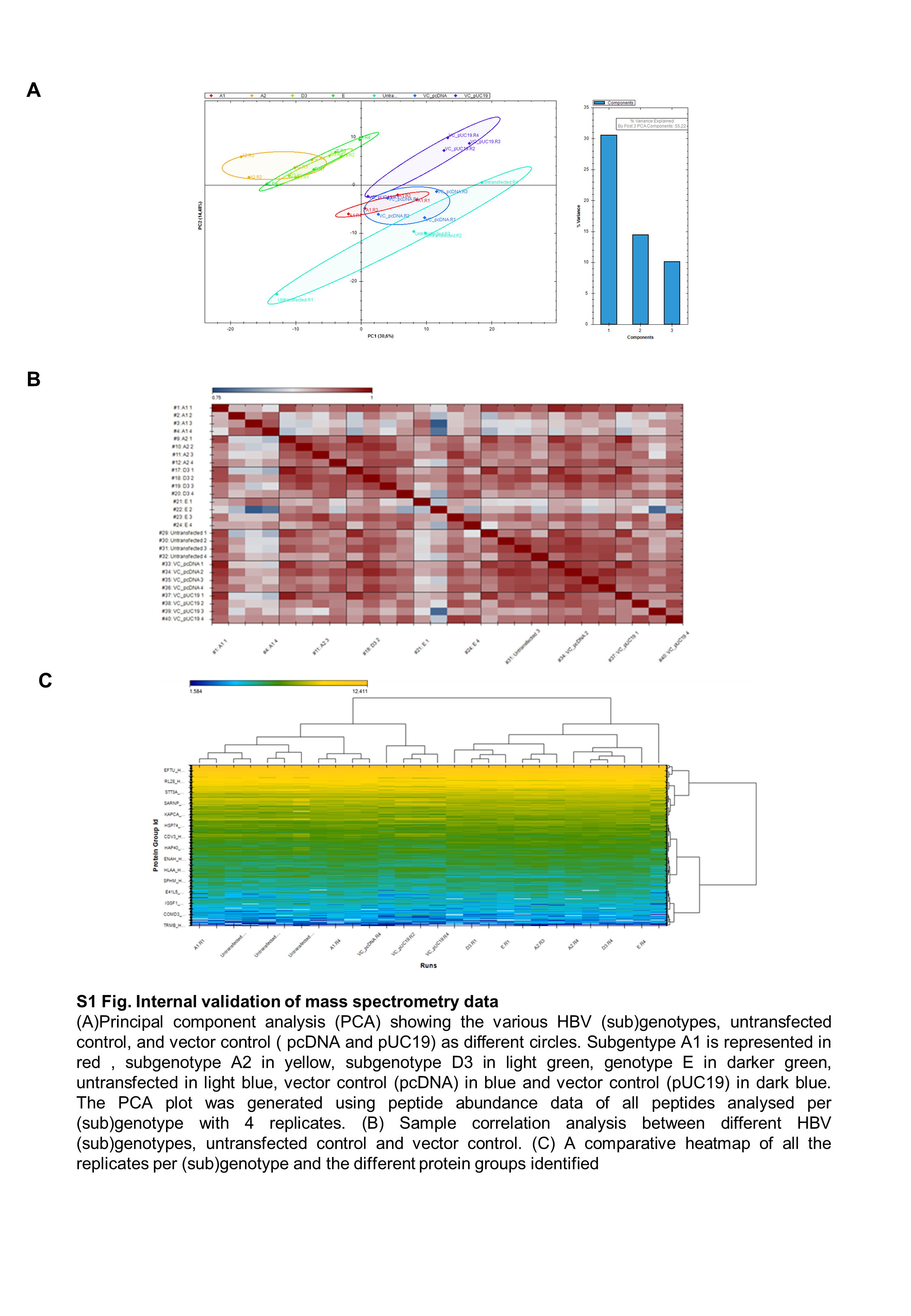

Supplement: Supplementary file 1 [file viruses-16-01052-s001.zip › S1.TIF]

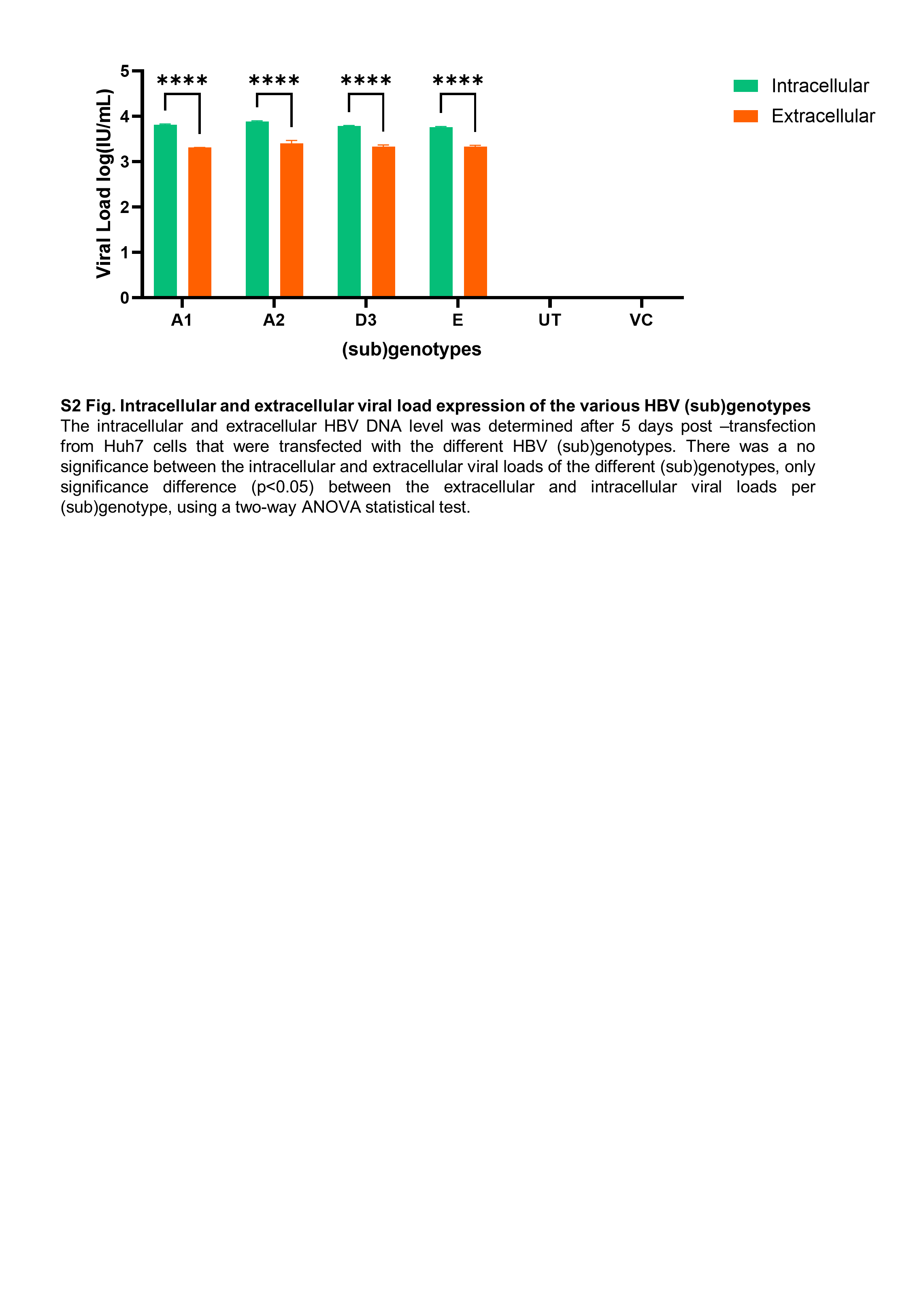

Supplement: Supplementary file 1 [file viruses-16-01052-s001.zip › S2.TIF]

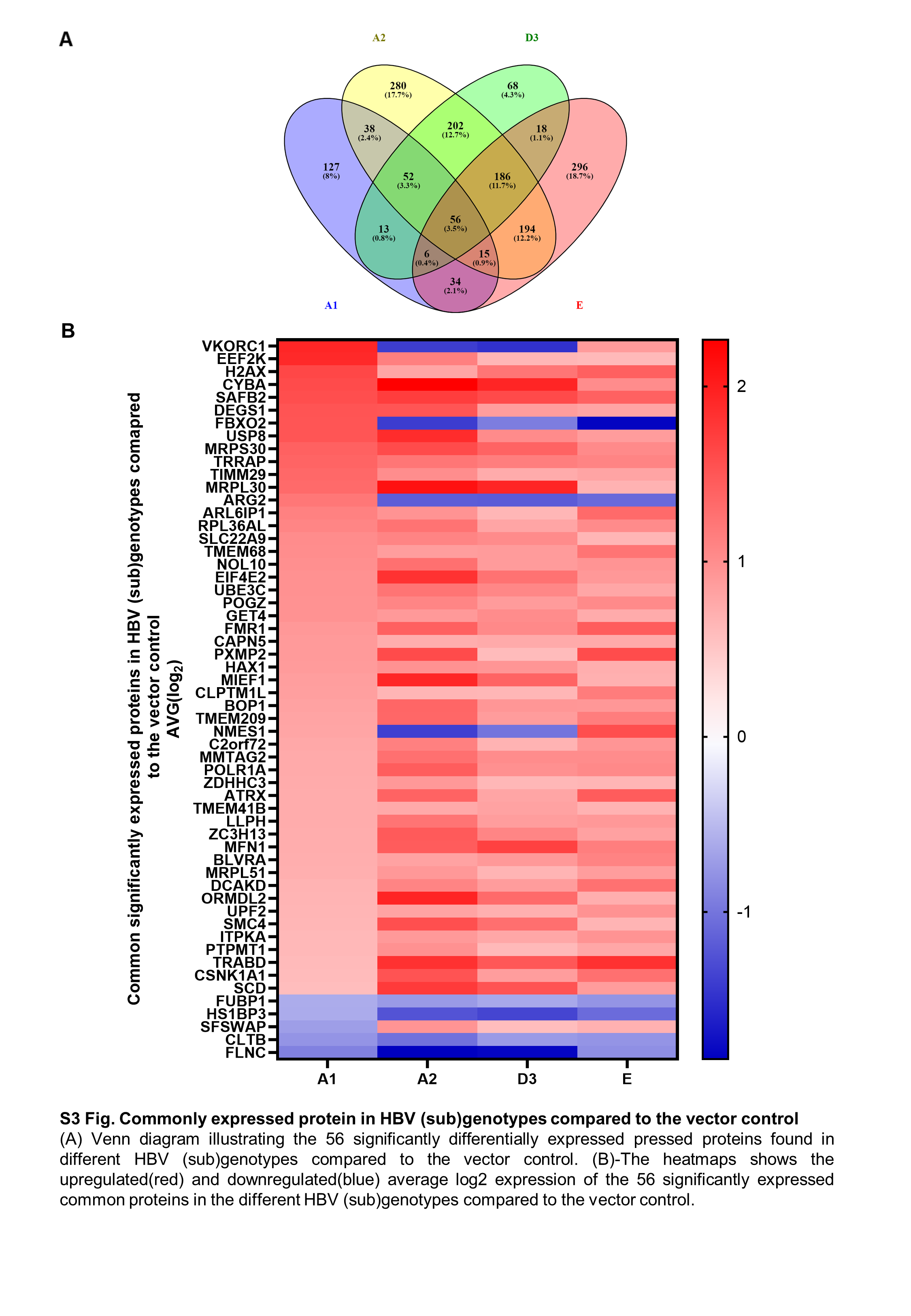

Supplement: Supplementary file 1 [file viruses-16-01052-s001.zip › S3.tif]
